# Long-Term Survival and Predictive Relevance of Nocturnal Ventricular Tachyarrhythmias Requiring Appropriate Implantable Cardioverter-Defibrillator Therapy

**DOI:** 10.31083/RCM51312

**Published:** 2026-10-16

**Authors:** Xuerong Sun, Xiaofeng Li, Shuang Zhao, Shu Zhang, Xingpeng Liu

**Affiliations:** ^1^Department of Cardiology, Heart Center and Beijing Key Laboratory of Hypertension, Beijing Chaoyang Hospital, Capital Medical University, 100020 Beijing, China; ^2^Arrhythmia Center, State Key Laboratory of Cardiovascular Disease, Fuwai Hospital, National Center for Cardiovascular Diseases, Chinese Academy of Medical Sciences and Peking Union Medical College, 100037 Beijing, China

**Keywords:** nocturnal, ventricular tachyarrhythmia, cardioverter-defibrillator therapy, mortality

## Abstract

**Background:**

Whether nocturnal ventricular tachyarrhythmias (VTAs) are associated with poorer long-term survival than daytime VTAs remains unclear. This study aimed to investigate the association between nocturnal VTAs and long-term survival, as well as the associated prognostic relevance.

**Methods:**

Implantable cardioverter-defibrillator (ICD) recipients from the Study of Home Monitoring System Safety and Efficacy in Cardiac Implantable Electronic Device-implanted Patients (SUMMIT) registry were retrospectively analyzed using data from home monitoring transmissions. Nocturnal VTAs were defined as episodes occurring between 22:00 and 05:59, whereas daytime VTAs were defined as those occurring during the remaining 16 hours (06:00–21:59).

**Results:**

Over a median follow-up of 61.7 months, 102 cardiac deaths and 187 deaths from any cause were recorded. Among 730 patients, 422 experienced VTAs, of whom 231 (54.7%) had nocturnal VTAs. Compared with patients without VTAs, those with nocturnal VTAs had a 62% higher risk of cardiac death (hazard ratio (HR), 1.62; 95% confidence interval (CI): 1.02–2.59; *p* = 0.041) and a 2.3-fold higher risk of all-cause mortality (HR, 2.30; 95% CI: 1.50–3.53; *p* < 0.001). Patients with nocturnal VTAs experienced more ICD shocks (77.9% [180/231] vs. 59.2% [113/191]; *p* < 0.001), more electrical storms (46.8% [108/231] vs. 9.4% [18/191]; *p* < 0.001), and a higher VTA burden (median, 1.36 [IQR, 3.44] vs. 0.59 [IQR, 1.01] episodes per year) than those with daytime-only VTAs. Elevated nighttime heart rate and reduced left ventricular ejection fraction (LVEF), as well as male sex and prior atrial fibrillation, were identified as independent risk factors for nocturnal VTAs.

**Conclusions:**

Nocturnal VTAs were not uncommon. Patients experiencing nocturnal VTAs had a higher risk of cardiac death and all-cause mortality. Monitoring the occurrence and timing of VTA episodes may be a useful tool for identifying high-risk patients.

**Clinical Trial Registration:**

ChiCTR-ONRC-13003695, https://www.chictr.org.cn/showproj.html?proj=5865.

## 1. Introduction

Life-threatening ventricular tachyarrhythmias (VTAs), including sustained ventricular tachycardia (VT) and ventricular fibrillation (VF), are a leading cause of sudden cardiac death (SCD) [1]. Survival rates following such events are generally poor, particularly for out-of-hospital cardiac arrest occurring at night [2,3]. In healthy individuals, nocturnal cardiovascular activity is predominantly modulated by the parasympathetic nervous system [4]. During rest, metabolism and endocrine function slow down, resulting in lower heart rate and blood pressure [5]. In the absence of physical and emotional stressors at night, the incidence of nocturnal VTAs would be expected to be low [6].

Previous studies have reported that the incidence of nocturnal SCD ranges from 17% to 41%, depending on the definition of nighttime hours [3,6]. Nevertheless, the pathogenesis of some nocturnal SCD cases remains unexplained [7]. In patients with an implantable cardioverter-defibrillator (ICD), lethal VTAs can be terminated by device-delivered therapies regardless of the time of day [1]. However, it remains unclear whether nocturnal VTAs that receive ICD therapy have a greater impact on patient survival than daytime VTAs.

In this study, we analysed the clinical relevance of VTA events in patients who underwent ICD or cardiac resynchronization therapy with defibrillator (CRT-D) implantation. The objectives were to describe the circadian distribution of the first VTA events post-implantation, evaluate the incidence of nocturnal VTAs and their association with long-term survival, and identify potential risk factors for nocturnal VTAs in ICD/CRT-D recipients.

## 2. Methods

### 2.1 Study Design and Participants

In the Study of Home Monitoring System Safety and Efficacy in Cardiac Implantable Electronic Device-implanted Patients (SUMMIT) registry (Registration number: ChiCTR-ONRC-13003695, https://www.chictr.org.cn/showproj.html?proj=5865), the patients receiving cardiac implantable electronic device (CIED) were prospectively enrolled from October 2013 to September 2014, including pacemaker, ICD, CRT, or CRT-D [8]. All patients enrolled at implantation time for their first device implant. Recruitment took place in 79 centres in China. Device implantations were carried out in line with established guideline [9] recommendations and incorporated a continuous home monitoring system (BIOTRONIK SE & Co. KG, Berlin, Germany). In the SUMMIT registry, the safety and efficacy of the home monitoring function were evaluated.

A retrospective analysis was performed on ICD/CRT-D recipients enrolled in the SUMMIT registry, based on their archived home monitoring transmission data. Patients were excluded from the analysis when one of the following conditions occurred: (1) unavailable or incomplete home monitoring data; (2) >10% of mean daily pacing (ventricular for single-chamber ICDs; atrial for dual-chamber ICDs) during the 30–60 day post-implant window; (3) less than 18 years of age at implantation; (4) lost to follow-up; (5) survived for less than 3 months after the device implantation; (6) diagnosed with malignancy; or (7) scheduled for heart transplantation. Approval for the present study was obtained from the Ethics Committees of the principal institution of Fuwai Hospital (the chief institute) and all collaborating sites. The study was carried out in line with the Declaration of Helsinki. Written informed consent was secured from each patient prior to enrollment.

### 2.2 Home Monitoring

Continuous home monitoring was initiated immediately after device implantation. Data were transmitted daily to the service centre via the antenna integrated into CIEDs, the CardioMessenger SMART (BIOTRONIK SE & Co. KG, Berlin, Germany), and the GSM network. To ensure complete home monitoring data capture, the CardioMessenger SMARTdevice needed to be maintained within an appropriate distance from the patient, remain adequately charged, and be kept free from electromagnetic interference. Additionally, the device required a stable connection to the GSM network to enable successful transmission of home monitoring data to the data processing centre.

The dataset from archived continuous home monitoring encompassed heart rate, pacing activity (atrial and/or ventricular), supraventricular tachyarrhythmias (e.g., atrial fibrillation [AF], atrial flutter, and sinus tachycardia), as well as VT/VF episodes, along with their corresponding therapies including anti-tachycardia pacing (ATP) and ICD shocks.

### 2.3 Follow-Ups and Study Endpoints

Routine follow-ups took place via home monitoring, telephone interviews, and clinic visits. In cases of transmission interruption for any reason, the research coordinator would promptly reach out to the patients or their family members to verify their health status, including any discomfort, hospitalizations, or death. Information on deaths and their dates was sourced from death certificates provided by family members. In this study, the primary endpoint was defined as cardiac death (ICD-10 codes: I00–I09, I11, I20–I51), while the secondary endpoint was all-cause mortality.

### 2.4 Device Programming

Device programming was as below: the base pacing rate was 40–60 bpm, and all patients received two therapy zones irrespective of the device type. The VT therapy zone was set at a heart rate of 170–209 bpm, and the detection duration was 26 beats with a 20-beat redetection; the VF therapy zone began at 210 bpm for faster tachycardia, and the detection duration was 12 out of 16 beats. The VT therapy included two to three attempts of ATP followed by cardioversion at progressively increasing energy. High-energy shocks were administered to terminate VF. During the follow-up, individual VT/VF therapy zones were only changed if there was a clinical indication. The intracardiac electrocardiograms (IEGMs) of VT/VF episodes were adjudicated in a blinded manner by two cardiologists. A determination was made to verify whether the documented event corresponded to VT/VF rather than supraventricular tachycardia, and an assessment was conducted regarding whether the patient received appropriate ICD therapy in the form of ATP and/or shock. When disagreement arose over interpretation of the IEGM or delivery of ICD therapy, a third cardiologist was consulted to render a final decision.

### 2.5 Definition of VTA Events

The VTA events were defined as the VT/VF episodes that received appropriate ATP and/or ICD shock following ICD/CRT-D implantation. Nocturnal VTAs were defined as VT/VF episodes that occurred between 22:00 and 05:59, and daytime VTAs were defined as VT/VF episodes that occurred during the remaining 16 hours of the day (between 06:00 and 21:59) over the whole follow-up period [6]. The presence and timing of the first nocturnal VTA events was acquired from the continuous home monitoring recording.

The VTA burden was defined as the number of VTA events occurring per year on average during the follow-up duration. Electrical storm was defined as the occurrence of at least three episodes of VTAs terminated by appropriate ICD therapies in a less than 24-hour period. An electrical storm was recorded as one VTA event when VTA burden was counted, and the start time was recorded as the electrical storm occurrence time.

### 2.6 Grouping

Based on the presence or absence of nocturnal VTA events alone over the whole follow-up period, the patients were further grouped into the no-VTA group, nocturnal VTA group, and only daytime VTA group.

### 2.7 Data Measurement and Collection

Nighttime heart rate was obtained via the home monitoring system when the patients were asleep. Nighttime heart rate was calculated as the mean heart rate between 02:00 and 06:00 at a time window of 30–60 days after the implantation procedure. To obtain accurate measured nighttime heart rate, mean daily pacing should be ≤10% (ventricular for single-chamber ICDs; atrial for dual-chamber devices).

Two senior echocardiography specialists measured the LVEF and left ventricular end-diastolic dimension (LVEDD) prior to device implantation (ICD or CRT-D). The LVEF values were subsequently calculated based on the modified biplane Simpson’s rule [10]. Information on other baseline characteristics, covering demographics, comorbid conditions, and prescribed medications, was extracted from patients’ medical records.

### 2.8 Statistical Analysis

Continuous data was presented as means ± standard deviations or medians (interquartile range, IQR) based on the metric of distribution; categorical data was presented as frequencies and percentages. One-way analyses of variance and Chi-square tests were used for continuous and categorical variables, respectively, to assess the differences between the three groups. VTA burden was involved into the statistical analysis as a categorical variable, which was described as low VTA burden (<1 episode per year) and high VTA burden (≥1 episode per year).

The Kaplan-Meier method was employed to evaluate differences in freedom from cardiac death and all-cause death across the different VTA groups. To evaluate the independent association of different types of VTAs with long-term cardiac death and all-cause mortality, three Cox regression models were constructed using the VTA groups as a time-varying covariate. And, given the competing risks, a Fine-Gray analysis was performed to evaluate the association between cardiac death and different types of VTAs. First, model 1 was established to explore the possible potential confounders using univariate Cox regression analysis. Second, potential confounders were involved in the multivariate Cox regression model 2, including age at implantation, LVEF, LVEDD, nighttime heart rate, CRT-D implantation, New York Heart Association (NYHA) class III–IV, hypertension, ischemic cardiomyopathy (ICM), prior myocardial infarction (MI), prior AF, use of angiotensin-converting enzyme inhibitors/angiotensin receptor blockers (ACEIs/ARBs), aldosterone antagonists, loop diuretics, digoxin, beta-blockers, and amiodarone. Third, multivariate Cox regression model 3 was adjusted for the above-mentioned confounders in model 2 and additional VTA burden.

In addition, univariate and multivariate Cox proportional hazards regression models were established to explore the potential risk factors for the first nocturnal VTA events after ICD/CRT-D implantation. Variables with a *p*-value ≤ 0.05 in univariable analysis were selected for testing in multivariable analysis.

Hazard ratios (HRs) and 95% confidence intervals (CIs) were calculated to determine the effect of nocturnal VTAs on mortality or each of the factors on the first nocturnal VTA events. Statistical significance was set at *p* < 0.05, and all tests were two-sided. All statistical analyses were carried out with SPSS Statistics (v23.0; IBM Corp., Armonk, NY, USA), R (v4.0.3; The R Foundation for Statistical Computing, Vienna, Austria), and GraphPad Prism (v9.0; GraphPad Software, La Jolla, CA, USA).

## 3. Results

### 3.1 Study Population and Baseline Characteristics

In brief, 730/1015 consecutive patients who underwent ICD or CRT-D implantation were enrolled. The remaining 285 patients were excluded due to >10% average daily atrial or ventricular pacing percentage (n = 50), unavailable or incomplete data (n = 227), age at implantation <18 years (n = 3), survival <3 months following device implantation (n = 3), and loss to follow-up (n = 2).

Comparisons of the baseline characteristics between the no-VTA group, nocturnal VTA group, and only daytime VTA group are summarised in Table 1. The overall mean age at implantation was 60.4 ± 13.9 years, and most patients were male (74.8%). ICD implantation was performed in 537 patients (73.6%) while the other 193 patients (26.4%) underwent CRT-D implantation. 305 patients (41.7%) received ICD or CRT-D implantation for primary prevention. Compared with the patients in the no-VTA and only daytime VTA groups, those in the nocturnal VTA group were mostly male (*p* = 0.048) with a higher proportion of patients had ICM (*p *= 0.044) and prior AF (*p *= 0.003).

**Table 1.** **Baseline characteristics**.

		Total	No-VTA group	Only daytime VTA group	Nocturnal VTA group	*p* value
		N = 730	n = 308	n = 191	n = 231	
Demographic characteristics						
	Age at implantation (years)	60.4 ± 13.9	60.9 ± 13.7	59.9 ± 15.2	60.1 ± 13.1	0.734
	Sex (male, n, %)	546 (74.8%)	218 (70.8%)	143 (74.9%)	185 (80.1%)	0.048
	BMI (kg/m^2^)	23.6 ± 3.0	23.5 ± 2.9	23.6 ± 3.0	23.5 ± 3.2	0.987
	CRT-D implantation (n, %)	193 (26.4%)	88 (28.6%)	44 (23.0%)	61 (26.4%)	0.395
	Primary prevention (n, %)	305 (41.7%)	136 (44.2%)	81 (42.4%)	88 (38.1%)	0.361
	NYHA class III–IV (n, %)	362 (49.6%)	148 (48.1%)	94 (49.2%)	120 (51.9%)	0.665
Echocardiographic characteristics						
	LVEF (%)	42.8 ± 14.9	44.3 ± 15.6	42.4 ± 14.7	41.2 ± 14.0	0.055
	LVEDD (mm)	58.7 ± 13.2	57.0 ± 12.4	60.2 ± 12.9	59.8 ± 14.3	0.011
Home monitoring data						
	Nighttime heart rate (bpm)	61.3 ± 9.1	61.3 ± 8.6	60.8 ± 8.5	61.8 ± 10.1	0.561
	24-hour heart rate (bpm)	70.9 ± 9.8	70.7 ± 9.4	71.1 ± 9.2	71.0 ± 10.8	0.912
Comorbidities						
	Hypertension (n, %)	228 (31.2%)	96 (31.2%)	54 (28.3%)	78 (33.8%)	0.479
	DM (n, %)	76 (10.4%)	30 (9.7%)	22 (11.5%)	24 (10.4%)	0.819
	Stroke (n, %)	16 (2.2%)	8 (2.6%)	4 (2.1%)	4 (1.7%)	0.789
	DCM (n, %)	171 (23.4%)	67 (21.8%)	45 (23.6%)	59 (25.5%)	0.589
	HCM (n, %)	29 (4.0%)	17 (5.5%)	7 (3.7%)	5 (2.2%)	0.138
	ICM (n, %)	247 (33.8%)	95 (30.8%)	59 (30.9%)	93 (40.3%)	0.044
	Prior MI (n, %)	102 (14.0%)	42 (13.6%)	25 (13.1%)	35 (15.2%)	0.811
	PCI (n, %)	65 (8.9%)	31 (10.1%)	14 (7.3%)	20 (8.7%)	0.573
	Prior AF (n, %)	83 (11.4%)	22 (7.1%)	23 (12.0%)	38 (16.5%)	0.003
	Preimplant presyncope (n, %)	41 (5.6%)	18 (5.8%)	8 (4.2%)	15 (6.5%)	0.577
	Preimplant syncope (n, %)	151 (20.7%)	56 (18.2%)	41 (21.5%)	54 (23.4%)	0.322
Medication						
	Beta-blockers (n, %)	413 (56.6%)	168 (54.5%)	109 (57.1%)	136 (58.9%)	0.597
	ACEI/ARBs (n, %)	257 (35.2%)	107 (34.7%)	66 (34.6%)	84 (36.4%)	0.905
	Aldosterone antagonists (n, %)	260 (35.6%)	108 (35.1%)	64 (33.5%)	88 (38.1%)	0.597
	CCBs (n, %)	61 (8.4%)	27 (8.8%)	14 (7.3%)	20 (8.7%)	0.836
	Statin (n, %)	169 (23.2%)	73 (23.7%)	41 (21.5%)	55 (23.8%)	0.813
	Loop diuretics (n, %)	189 (25.9%)	80 (26.0%)	44 (23.0%)	65 (28.1%)	0.492
	Digoxin (n, %)	141 (19.3%)	60 (19.5%)	32 (16.8%)	49 (21.2%)	0.511
	Amiodarone (n, %)	212 (29.0%)	87 (28.2%)	50 (26.2%)	75 (32.5%)	0.338
	Antiplatelet (n, %)	152 (20.8%)	67 (21.8%)	29 (15.2%)	56 (24.2%)	0.064

ACEIs, angiotensin-converting enzyme inhibitors; AF, atrial fibrillation; ARBs, angiotensin receptor blockers; BMI, body mass index; bpm, beats per minute; CCB, calcium channel blockers; ICD, implantable cardioverter-defibrillator; DCM, dilated cardiomyopathy; DM, diabetes mellitus; HCM, hypertrophic cardiomyopathy; LVEDD, left ventricular end-diastolic dimension; LVEF, left ventricular ejection fraction; MI, myocardial infarction; PCI, percutaneous coronary intervention; VTA, ventricular tachyarrhythmia; CRT-D, cardiac resynchronization therapy with defibrillator; NYHA, New York Heart Association; ICM, ischemic cardiomyopathy.

### 3.2 Circadian Distribution of the First VTA Events

During a median follow-up of 61.7 (26.4) months, 422 patients (57.8%) experienced at least one VTA event that received appropriate cardioverter-defibrillator therapy. For the first VTA events following implantation, 105 episodes (average, 13.1 episodes per hour) were observed during the nighttime and 317 episodes (average, 19.8 episodes per hour) during the daytime. The circadian distribution of the first VTA events during the 24-hour period are depicted in Fig. 1. A trough during the nighttime hours (22:00–05:59) and two prominent peaks—one in the morning (07:00–10:00) and one after noon (13:00–15:00)—were noted (Fig. 1).

**Fig. 1.**
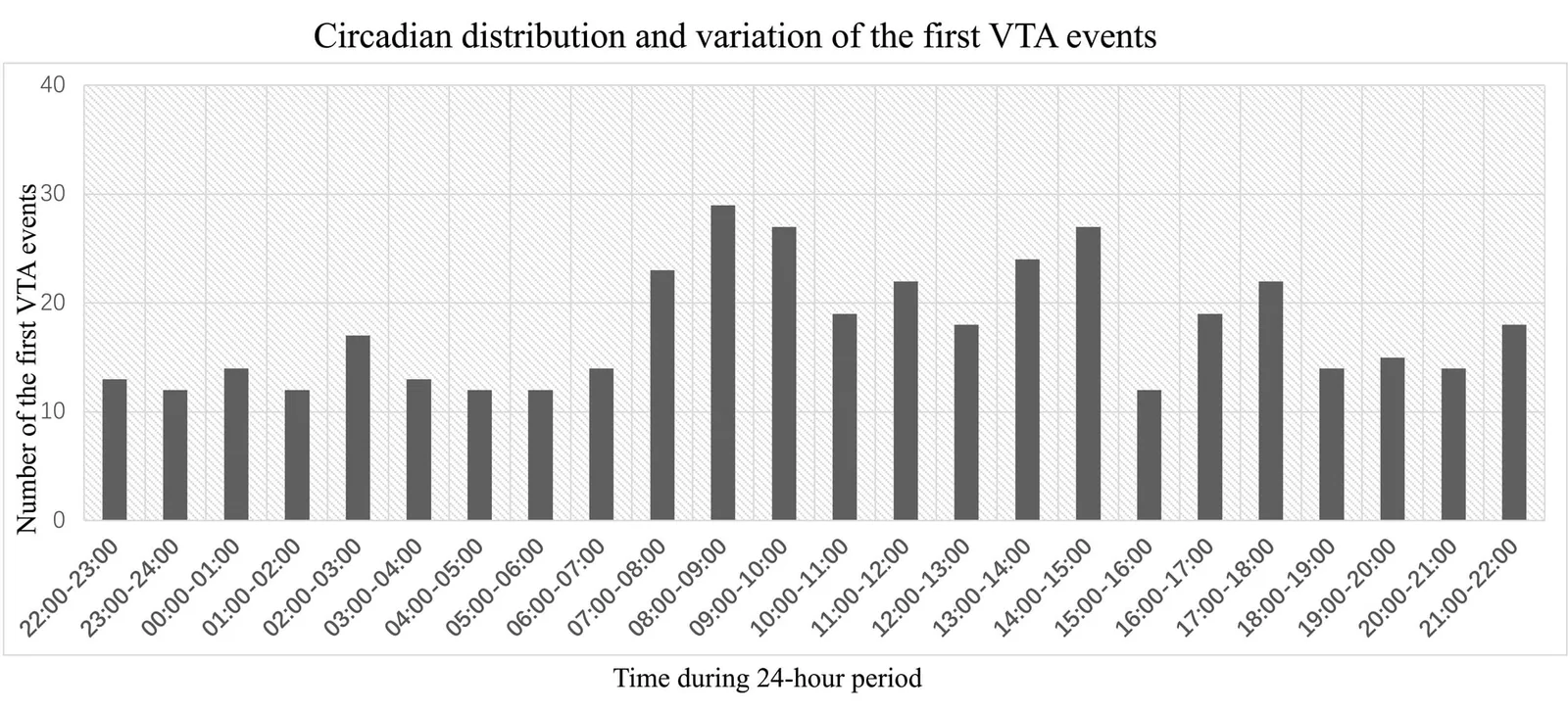
**The circadian distribution and variation of the first VTA events post device implantation**. The circadian distribution of the first VTA events during the 24-hour period are depicted in Fig. 1. A trough during the nighttime hours (22:00–05:59) and two prominent peaks—one in the morning (07:00–10:00) and one after noon (13:00–15:00)—were noted.

### 3.3 Incidence of Nocturnal VTAs, Cardiac Death and All-Cause Death

Over 5.1 years, of 422 patients with incident VTAs, 231 patients (54.7%) experienced nighttime VTAs with or without daytime VTAs (nocturnal VTA group, n = 231) and 191 patients (45.2%) experienced only daytime VTAs (only daytime group, n = 191). 102 cardiac deaths (14.0%) and 187 deaths from any cause (25.6%) were observed.

### 3.4 Electrical Storm and VTA Burden

More patients in the nocturnal VTA group suffered from appropriate ICD shocks (77.9% [180/231] vs. 59.2% [113/191], respectively, *p *< 0.001) and electrical storm (46.8% [108/231] vs. 9.4% [18/191], respectively, *p *< 0.001) than those in the only daytime VTA group.

The VTA burden in the nocturnal VTA group (median, 1.36 [IQR, 3.44] episodes per year) was heavier than that in the only daytime VTA group (median, 0.59 [IQR, 1.01] episode per year). The proportion of the patients with high VTA burden (≥1 episode per year) in the nocturnal VTA group and the only daytime VTA group was 56.3% and 31.4%, respectively.

### 3.5 Nocturnal VTAs Associated With Long-Term Survival

More patients suffered cardiac death in the nocturnal VTA group than that in the only daytime VTA and no-VTA groups (24.2% vs. 13.6% vs. 6.5%, respectively). Similarly, the incidence of death from any cause was significantly higher in the nocturnal VTA group than that in the other two groups (38.5% vs. 22.0% vs. 18.2%, respectively) (Table 2).

**Table 2.** **Cox regression analysis for long-term mortality**.

		No. of cases (n)	No. of death (n, %)	Univariate Cox regression analysis (Model 1)		Multivariate Cox regression analysis (Model 2)		Multivariate Cox regression analysis (Model 3)	
				HR (95% CI)	*p* value	HR (95% CI)	*p* value	HR (95% CI)	*p* value
Cardiac death		730	102 (14.0%)						
	No-VTA	308	20 (6.5%)	Ref.		Ref.		Ref.	
	Only daytime VTA	191	26 (13.6%)	2.10 (1.17–3.76)	*p* = 0.013	1.99 (1.02–3.87)	*p* = 0.044	1.39 (0.75–2.57)	*p* = 0.285
	Nocturnal VTA	231	56 (24.2%)	3.74 (2.24–6.23)	*p* < 0.001	2.15 (1.19–3.90)	*p* = 0.011	1.62 (1.02–2.59)	*p* = 0.041
All-cause death		730	187 (25.6%)						
	No-VTA	308	56 (18.2%)	Ref.		Ref.		Ref.	
	Only daytime VTA	191	42 (22.0%)	1.37 (0.89–2.10)	*p* = 0.153	1.32 (0.85–2.05)	*p* = 0.215	1.23 (0.78–1.94)	*p* = 0.376
	Nocturnal VTA	231	89 (38.5%)	2.56 (1.78–3.69)	*p* < 0.001	2.58 (1.78–3.76)	*p* < 0.001	2.30 (1.50–3.53)	*p* < 0.001

Multivariate Cox regression model 2 was adjusted for age at implantation, LVEF, LVEDD, nighttime heart rate, CRT-D implantation, NYHA class III–IV, hypertension, ICM, prior MI, prior AF, the use of ACEI/ARBs, aldosterone antagonists, loop diuretics, digoxin, beta-blockers, and amiodarone. Multivariate Cox regression model 3 was adjusted for the above-mentioned confounders in model 2 and additional VTA burden. ACEIs, angiotensin-converting enzyme inhibitors; AF, atrial fibrillation; ARBs, angiotensin receptor blockers; CI, confidence interval; CRT-D, cardiac resynchronization therapy defibrillator; HR, hazard ratio; ICM, ischemic cardiomyopathy; LVEDD, left ventricular end-diastolic dimension; LVEF, left ventricular ejection fraction; MI, myocardial infarction; VTA, ventricular tachyarrhythmia.

Kaplan-Meier survival curves were demonstrated that the patients who experienced nocturnal VTAs had significant higher incidence of cardiac death and all-cause mortality in Fig. 2.

**Fig. 2.**
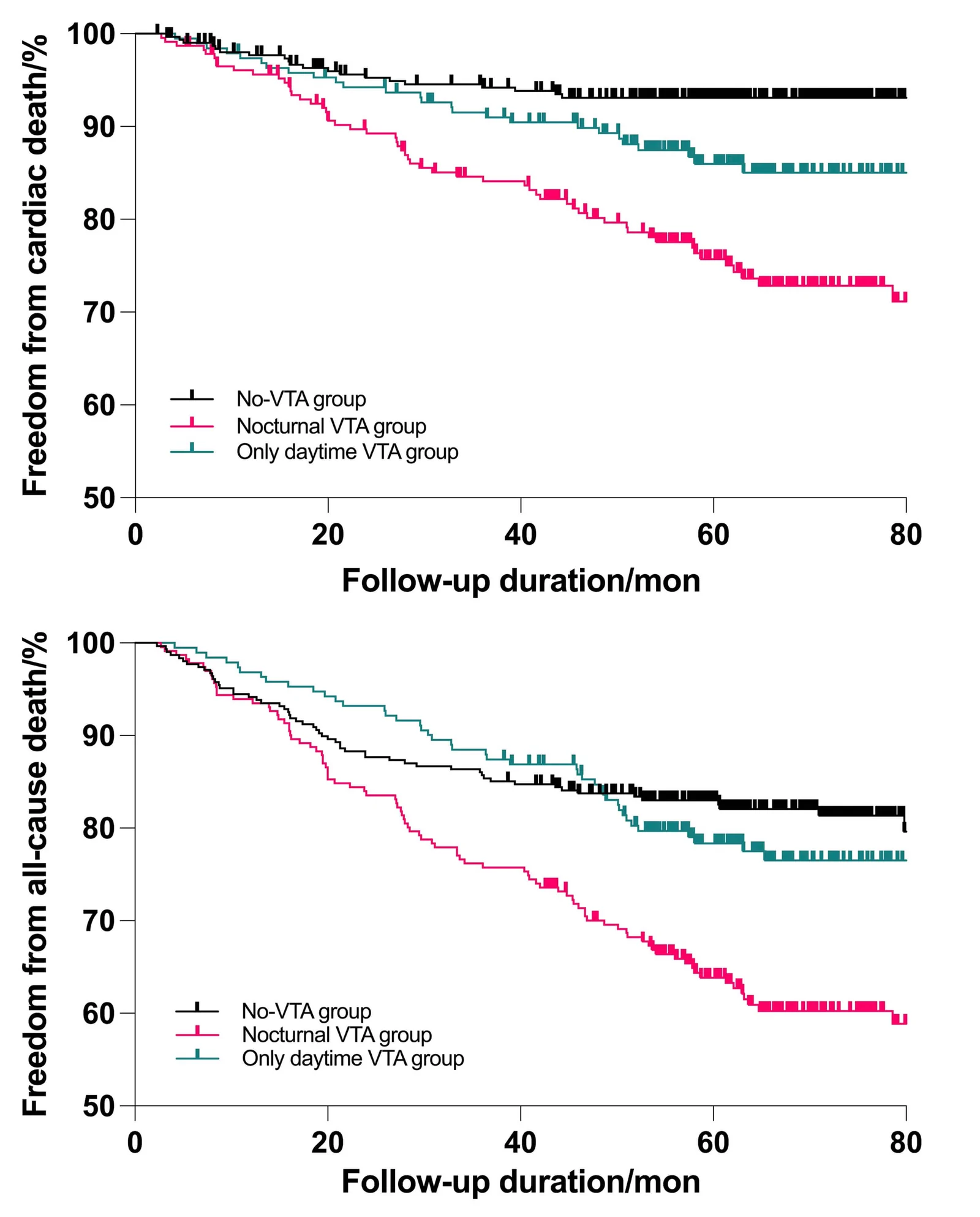
**Kaplan-Meier survival analysis of cardiac death and all-cause mortality**. Kaplan-Meier survival curves were demonstrated that the patients who experienced nocturnal VTAs had higher incidence of cardiac death and all-cause mortality in Fig. 2. VTA, ventricular tachyarrhythmia.

### 3.6 Negative Effects of Nocturnal VTAs on Prognosis

Regarding cardiac death, in the univariate Cox regression analysis, both patients in the nocturnal VTA group (HR, 3.74; 95% CI: 2.24–6.23; *p *< 0.001) and patients in the only daytime VTA group (HR, 2.10; 95% CI: 1.17–3.76; *p *= 0.013) had a significantly higher mortality than those without observed VTA events (Table 2). The results remained consistent in the multivariate Cox regression model 2, which was adjusted for the potential confounding factors. The patients who experienced nocturnal VTAs (HR, 2.15; 95% CI: 1.19–3.90; *p *= 0.011) and only daytime VTAs (HR, 1.99; 95% CI: 1.02–3.87; *p *= 0.044) had 2.2-fold and 2.0-fold higher risks of cardiac death than those who did not experienced VTAs after ICD/CRT-D implantation, respectively (Table 2). After adjusted for additional VTA burden, nocturnal VTA was still associated with a significantly higher risk of cardiac death (HR, 1.62; 95% CI: 1.02–2.59; *p *= 0.041), while the only daytime VTA group was not (HR, 1.39; 95% CI: 0.75–2.57; *p *= 0.285) (Table 2). Besides this, high VTA burden was shown associated with 75% increased risks of cardiac death (HR, 1.75; 95% CI: 1.09–2.92; *p *= 0.026).

For all-cause death, patients who experienced nocturnal VTAs had a significantly higher all-cause mortality (HR, 2.56; 95% CI: 1.78–3.69; *p *< 0.001) than those without observed VTA events, while the patients who only experienced daytime VTAs did not (HR, 1.37; 95% CI: 0.89–2.10; *p *= 0.153) (Table 2) in the univariate analysis. In the multivariate Cox regression model 2, the results were similar. The nocturnal VTA group had a 2.6-fold higher risk of all-cause mortality when compared with the no-VTA group (HR, 2.58; 95% CI: 1.78–3.76; *p *< 0.001). Only the daytime VTA group did not have a significantly increased incidence of all-cause mortality (HR, 1.32; 95% CI: 0.85–2.05; *p *= 0.215) (Table 2). After adjusted for additional VTA burden, the results remained consistent (Table 2). In addition, no significant association was shown between all-cause mortality and VTA burden (HR, 1.25; 95% CI: 0.85–1.84; *p *= 0.251).

### 3.7 Predictive Relevance of Nocturnal VTAs

Univariable Cox regression analysis revealed that elevated nighttime heart rate, reduced LVEF, male sex, and prior AF were associated with a significantly increased risk of nocturnal VTAs (Table 3). In the multivariable Cox regression model adjusted for nighttime heart rate, LVEF, male sex, and prior AF, nighttime heart rate ≥66.4 bpm (Quantile 4) (HR, 1.37; 95% CI: 1.03–1.83; *p *= 0.031), LVEF ≤55% (Quantile 1–3) (HR, 1.42; 95% CI: 1.02–1.97; *p *= 0.039), male sex (HR, 1.51; 95% CI: 1.09–2.08; *p *= 0.013), and prior AF (HR, 1.60; 95% CI: 1.12–2.27; *p *= 0.009) were demonstrated to be independent risk predictors of nocturnal VTAs; furthermore, these factors resulted in 37%, 42%, 51%, and 60% increased risks of nocturnal VTAs, respectively (Table 3).

**Table 3.** **Cox regression analysis for the first nocturnal VTAs**.

		HR (95% CI)	*p* value
Univariable Cox regression analysis			
	Nighttime heart rate^†^	1.02 (1.00–1.03)	*p* = 0.050
	Nighttime heart rate Q1–3: Q4	1.47 (1.11–1.96)	*p* = 0.008
	LVEF^*^	1.01 (1.00–1.02)	*p* = 0.010
	LVEF Q4: Q1–3	1.92 (1.14–3.24)	*p* = 0.014
	Male, sex	1.54 (1.12–2.13)	*p* = 0.009
	Prior AF	1.73 (1.22–2.45)	*p* = 0.002
Multivariable Cox regression analysis			
	Nighttime heart rate Q1–3: Q4	1.37 (1.03–1.83)	*p* = 0.031
	LVEF Q4: Q1–3	1.42 (1.02–1.97)	*p* = 0.039
	Male, sex	1.51 (1.09–2.08)	*p* = 0.013
	Prior AF	1.60 (1.12–2.27)	*p* = 0.009

Multivariable Cox regression model was adjusted for nighttime heart rate, LVEF, male sex and prior AF. AF, atrial fibrillation; CI, confidence interval; HR, hazard ratio; LVEF, left ventricular ejection fraction; VTA, ventricular tachyarrhythmia. ^†^ increase/per unit; * decrease/per unit.

## 4. Discussion

The present study obtained detailed information on the presence and timing of VT/VF episodes and ICD therapy along with other useful data through the continuous home monitoring system, which provided important insights regarding the clinical relevance of nocturnal VTAs in ICD/CRT-D recipients. First, the incidence of nocturnal VTAs was shown not low. 231/730 patients experienced nocturnal VTAs over 5.1 years, which accounted for 54.7% (231/422) of the patients with observed VTAs. Second, the time-dependent modelling incorporating multiple VTA types demonstrated nocturnal VTAs were associated with higher risks of the subsequent death than daytime VTAs. Compared with no-VTAs, nocturnal VTAs were associated with a 62% higher risk of cardiac death and a 2.3-fold higher risk of all-cause mortality. In contrast, patients who only experienced daytime VTAs had no significantly increased risks of cardiac death and all-cause mortality. Third, patients in the nocturnal VTA group suffered from more ICD shocks, more electrical storms, and heavier VTA burden than those in the only daytime VTA group. Multivariable Cox regression analysis showed that elevated nighttime heart rate, reduced LVEF, male sex, and prior AF were independent predictors of nocturnal VTAs, resulting in 37%, 42%, 51%, and 60% increased risks of nocturnal VTAs, respectively.

A clear circadian pattern emerges for both SCD and episodes of VT/VF, with events clustering significantly during the early morning hours after waking, which was consistent with our results [4]. The circadian distribution of the first VTA events showed a prominent peak in the morning (07:00–10:00). This circadian rhythm may manifest as a daily variation in a proarrhythmic trigger—specifically, a morning surge in sympathetic drive. β-adrenergic stimulation promotes Ca^2+^ overload, delayed afterdepolarizations, and reentry [4]. However, during the night, the parasympathetic activity was predominant and the incidence of nocturnal VTAs might be low. Our analysis showed during the nighttime, 13.1 episodes per hour was observed, in comparison to 19.8 episodes per hour during the daytime. In addition, more than half patients who experienced VTA events suffered from nocturnal VTAs. Thus, the incidence of nocturnal VTAs was not as low as expected. Exploring the effects of nocturnal VTAs on the long-term prognosis in ICD/CRT-D recipients might bring some clinically important insights.

In previous studies, in-hospital cardiac arrest events occurred during both the nighttime and daytime hours, which resulted in different short-term survival outcomes following SCA [3,11,12,13]. Patients who suffered from nocturnal in-hospital SCA had worse clinical outcomes in terms of the restoration of spontaneous circulation, survival-to-hospital discharge, 24-hour survival, and neurological disabilities over time when compared with those with daytime SCA [11,12]. The poor survival outcomes of in-hospital SCA might be attributed to the delayed detection and recognition of VTAs, fewer nurses, and low-quality cardiopulmonary resuscitation during the nighttime hours [12]. Wallace et al. [13] discovered that in patients who experienced out-of-hospital cardiac arrest, 30-day survival post-SCA was significantly lower at night than that during the day after adjusting for the characteristics of patients and events and differences in prehospital care. The low survival rate following out-of-hospital SCA may be explained by the rapidly evolving urgency of cardiac arrest and the critical role of immediate bystander resuscitation [3,13]. In ICD/CRT-D recipients, although VTAs can be terminated by cardioverter-defibrillator therapies, the effects of the circadian variation in VTAs on the long-term survival have not been explored previously. The findings of this study demonstrated that patients who experienced nighttime VTAs had a 62% higher risk of cardiac death and a 2.3-fold higher risk of all-cause mortality over 5.1 years when compared with those without observed VTA events; however, daytime VTAs did not have higher risk of cardiac death, appearing not to threaten the long-term survival in ICD/CRT-D recipients. Patients in the nocturnal VTA group had heavier VTA burden than those in the only daytime VTA group (1.36 vs. 0.59 episode per year). After the multivariate Cox regression models were adjusted for additional VTA burden, the association between nocturnal VTAs and worse long-term survival was still robust. This major finding is novel and highlights the importance of paying close attention to the careful monitoring and timely management of nocturnal VTAs following ICD/CRT-D implantation.

The observed contribution of nocturnal VTAs to worse long-term survival might be explained by the possibility that these arrhythmias reflect, at least in part, the severity of the underlying disease. In our study, patients who experienced nocturnal VTAs were more likely to receive appropriate ICD shocks (77.9% vs. 59.2%, *p* < 0.001) and electrical storms (46.8% vs. 9.4%, *p* < 0.001) than those who did not experience nocturnal VTAs. This evidence highlights that nocturnal VTAs could include more shock namely VF episodes, faster VT episodes, or VT episodes which are recalcitrant to ATP therapies [14]. VTA events that occur during the night are expected to be more life-threatening and hemodynamically unstable, probably because nocturnal VTAs were related to worse ventricular myocardial substrate [15]. The SCD-HeFT study reported that ICD recipients had a four-fold increase in the risk of death from any cause after receiving an appropriate ICD shock [14].

Furthermore, the adverse prognostic significance of nocturnal VTAs may be explained by their independent pathophysiological role. In this context, we found that nighttime heart rate, LVEF, prior AF, and male sex were independent risk factors for the occurrence of nocturnal VTAs. First, nighttime heart rate was measured during the sleeping periods (02:00–06:00), which minimised the interference due to sensory inputs as well as physical and mental activities [16,17]. Thus, nighttime heart rate could reflect the intrinsic cardiac autonomic function in modulating the cardiovascular activity [16]. High levels of nighttime heart rate indicated increased sympathetic activity [16]. elevated nighttimeHeart rate was associated with poor physical conditions—such as heightened oxidative stress, increased arterial stiffness, impaired left ventricular performance, endothelial dysfunction, systemic inflammatory responses, plaque disruption, and elevated myocardial oxygen demand—which are detrimental to long-term survival [17]. Second, heart failure (HF) with lower LVEF often coexists with AF, and both are responsible for increased mortality rates [18]. AF is associated with more VTAs and appropriate and inappropriate ICD shocks, especially in patients with HF [19]. During an atrial tachyarrhythmia, the rapid ventricular rate might reduce the ventricular refractoriness and worsen the electrical instability of the ventricular myocardium [20]. The hemodynamic effects of rapid and irregular atrial mechanical dysfunction may result in neurohumoral activation of the sympathetic tone [20]. Third, male patients with ICD/CRT-D implantation probably are more likely to experience nocturnal VTA events, which reflects the gender differences in SCD in the general population [21]. The incidence of out-of-hospital cardiac arrest was approximately three-fold higher in men than that in women [22]. This might be linked to more structural heart diseases, such as ICM, hypertrophic cardiomyopathy, and dilated cardiomyopathy, which are diagnosed before SCD in men [23]. Also, some cardiomyopathy-associated gene variants are not only more prevalent in men but also result in peak VTAs during the nighttime hours [6,7,24].

Therefore, the underlying mechanisms by which nocturnal VTAs contribute to worse long-term survival are multifactorial, comprehensive, and probably include various aspects of cardiac autonomic nervous function and structural and electrical remodelling.

The clinical implications of this study are that to improve the long-term survival in patients with high risks of SCD, only providing appropriate ICD therapies is not enough. We have identified time-dependent markers of subsequent increased mortality risk, namely, nocturnal VTA events. Monitoring the presence and timing of VT/VF episodes might be a useful tool in identifying high-risk patients. The possible mechanisms underlying the association of nocturnal VTAs with subsequent mortality was indicated, including sympathetic activity, worsening HF, incident AF episodes, etc. It is possible that reevaluation of cardiovascular status and optimization of therapies, especially for improving HF, modulating cardiac autonomic function, and reducing AF episodes, may be considered in this group of patients. Also, ablation of the underlying atrial or ventricular proarrhythmic substrate may be considered when possible.

## 5. Limitation

First, patients who experienced nocturnal VTAs suffered from heavier VTA burden than those who only experienced daytime VTA events (1.36 vs. 0.59 episode per year). However, the Cox regression models, established to explore the effects of the VTA types on cardiac death and all-cause mortality, was adjusted for VTA burden. Due to the small sample, it was hard to make the direct comparison between patients who only experienced nocturnal or daytime VTAs. Thus, the potential impact of VTA burden on death was still indefinite. Second, sleep-disordered breathing (SDB) is a very common and frequently underdiagnosed comorbidity in patients with HF and AF [25,26]. Nocturnal VTAs are prevalent in patients with SDB [27]. In this analysis, reduced LVEF and AF were demonstrated as risk factors of mortality; however, the data regarding SDB is not obtained. Thus, without adjusting for this, the causal interpretation may be weakened. Third, compared to the recommended tachyarrhythmia detection duration criteria [28], which was at least 6 seconds or for 30 beats before completing detection, the programmed detection duration (26 beats) in this cohort study was shorter. Some clinically irrelevant VTA events were possibly included. Fourth, additional prospective research is warranted to elucidate the causal relationship between the circadian rhythm of VTAs and long-term survival. Fifth, selection bias may be introduced by excluding patients who died within the first 3 months post-implantation.

## 6. Conclusions

Nocturnal VTAs were associated with significantly increased risks of both cardiac death and all-cause mortality over five years. Notably, over half of the patients who experienced VTAs had nocturnal episodes. Monitoring the presence and timing of VT/VF episodes may serve as a useful tool for identifying high-risk patients.

## Data Availability

The datasets generated and analysed during the current study are not publicly available due to the Fuwai Hospital regulations but are available from the corresponding authors on reasonable request.
